# Supplementary material for: Resolving the full spectrum of human genome variation using Linked-Reads
Source: Genome Res. 2019 Apr;29(4):635–45. doi: 10.1101/gr.234443.118 (PMC6442396; doi:10.1101/gr.234443.118)
Supplement: Supplemental Material [file supp_gr.234443.118_Supplemental_longranger-2.2.zip › longranger-2.2.tar.bz2!/lr2.2-public/lib/stan/cmdstan/stan/doc/api/html/namespacestan_1_1optimization.html]

Stan: stan::optimization Namespace Reference


|  |  |
| --- | --- |
|  | Stan  2.5.0  probability, sampling & optimization |


- Main Page
- Related Pages
- Namespaces
- Classes
- Files

- Namespace List
- Namespace Members

All Classes Namespaces Files Functions Variables Typedefs Enumerations Enumerator Friends Macros Pages

- stan
- optimization

Classes |
Typedefs |
Enumerations |
Functions

stan::optimization Namespace Reference

|  |  |
| --- | --- |
| Classes | |
| class | ConvergenceOptions |
|  | |
| class | LSOptions |
|  | |
| class | BFGSMinimizer |
|  | |
| class | ModelAdaptor |
|  | |
| class | BFGSLineSearch |
|  | |
| class | BFGSUpdate\_HInv |
|  | |
| class | LBFGSUpdate |
|  | Implement a limited memory version of the BFGS update. More... |
|  | |

|  |  |
| --- | --- |
| Typedefs | |
| typedef Eigen::Matrix< double,   Eigen::Dynamic, Eigen::Dynamic > | matrix\_d |
|  | |
| typedef Eigen::Matrix< double,   Eigen::Dynamic, 1 > | vector\_d |
|  | |

|  |  |
| --- | --- |
| Enumerations | |
| enum | TerminationCondition {     TERM\_SUCCESS = 0, TERM\_ABSX = 10, TERM\_ABSF = 20, TERM\_RELF = 21,     TERM\_ABSGRAD = 30, TERM\_RELGRAD = 31, TERM\_MAXIT = 40, TERM\_LSFAIL = -1   } |
|  | |

|  |  |
| --- | --- |
| Functions | |
| template<typename M > | |
| double | lp\_no\_jacobian (const M &model, std::vector< double > &params\_r, std::vector< int > &params\_i, std::ostream \*o=0) |
|  | |
| template<typename Scalar > | |
| Scalar | CubicInterp (const Scalar &df0, const Scalar &x1, const Scalar &f1, const Scalar &df1, const Scalar &loX, const Scalar &hiX) |
|  | Find the minima in an interval [loX, hiX] of a cubic function which interpolates the points, function values and gradients provided. More... |
|  | |
| template<typename Scalar > | |
| Scalar | CubicInterp (const Scalar &x0, const Scalar &f0, const Scalar &df0, const Scalar &x1, const Scalar &f1, const Scalar &df1, const Scalar &loX, const Scalar &hiX) |
|  | Find the minima in an interval [loX, hiX] of a cubic function which interpolates the points, function values and gradients provided. More... |
|  | |
| template<typename FunctorType , typename Scalar , typename XType > | |
| int | WolfeLineSearch (FunctorType &func, Scalar &alpha, XType &x1, Scalar &f1, XType &gradx1, const XType &p, const XType &x0, const Scalar &f0, const XType &gradx0, const Scalar &c1, const Scalar &c2, const Scalar &minAlpha) |
|  | Perform a line search which finds an approximate solution to:   satisfying the strong Wolfe conditions: 1)  2)  where  is the gradient of f(x). More... |
|  | |
| template<typename M > | |
| double | newton\_step (M &model, std::vector< double > &params\_r, std::vector< int > &params\_i, std::ostream \*output\_stream=0) |
|  | |

## Typedef Documentation

typedef Eigen::Matrix<double, Eigen::Dynamic, Eigen::Dynamic> stan::optimization::matrix\_d

Definition at line 14 of file newton.hpp.

typedef Eigen::Matrix<double, Eigen::Dynamic, 1> stan::optimization::vector\_d

Definition at line 15 of file newton.hpp.

## Enumeration Type Documentation

enum stan::optimization::TerminationCondition

| Enumerator | |
| --- | --- |
| *TERM\_SUCCESS* |  |
| *TERM\_ABSX* |  |
| *TERM\_ABSF* |  |
| *TERM\_RELF* |  |
| *TERM\_ABSGRAD* |  |
| *TERM\_RELGRAD* |  |
| *TERM\_MAXIT* |  |
| *TERM\_LSFAIL* |  |

Definition at line 21 of file bfgs.hpp.

## Function Documentation

template<typename Scalar >

|  |  |  |  |
| --- | --- | --- | --- |
| Scalar stan::optimization::CubicInterp | ( | const Scalar & | *df0*, |
|  |  | const Scalar & | *x1*, |
|  |  | const Scalar & | *f1*, |
|  |  | const Scalar & | *df1*, |
|  |  | const Scalar & | *loX*, |
|  |  | const Scalar & | *hiX* |
|  | ) |  |  |

Find the minima in an interval [loX, hiX] of a cubic function which interpolates the points, function values and gradients provided.

Implicitly, this function constructs an interpolating polynomial g(x) = a\_3 x^3 + a\_2 x^2 + a\_1 x + a\_0 such that g(0) = 0, g(x1) = f1, g'(0) = df0, g'(x1) = df1 where g'(x) = 3 a\_3 x^2 + 2 a\_2 x + a\_1 is the derivative of g(x). It then computes the roots of g'(x) and finds the minimal value of g(x) on the interval [loX,hiX] including the end points.

This function implements the full parameter version of CubicInterp().

Parameters
:   |  |  |
    | --- | --- |
    | df0 | First derivative value, f'(x0) |
    | x1 | Second point |
    | f1 | Second function value, f(x1) |
    | df1 | Second derivative value, f'(x1) |
    | loX | Lower bound on the interval of solutions |
    | hiX | Upper bound on the interval of solutions |

Definition at line 35 of file bfgs\_linesearch.hpp.

template<typename Scalar >

|  |  |  |  |
| --- | --- | --- | --- |
| Scalar stan::optimization::CubicInterp | ( | const Scalar & | *x0*, |
|  |  | const Scalar & | *f0*, |
|  |  | const Scalar & | *df0*, |
|  |  | const Scalar & | *x1*, |
|  |  | const Scalar & | *f1*, |
|  |  | const Scalar & | *df1*, |
|  |  | const Scalar & | *loX*, |
|  |  | const Scalar & | *hiX* |
|  | ) |  |  |

Find the minima in an interval [loX, hiX] of a cubic function which interpolates the points, function values and gradients provided.

Implicitly, this function constructs an interpolating polynomial g(x) = a\_3 x^3 + a\_2 x^2 + a\_1 x + a\_0 such that g(x0) = f0, g(x1) = f1, g'(x0) = df0, g'(x1) = df1 where g'(x) = 3 a\_3 x^2 + 2 a\_2 x + a\_1 is the derivative of g(x). It then computes the roots of g'(x) and finds the minimal value of g(x) on the interval [loX,hiX] including the end points.

Parameters
:   |  |  |
    | --- | --- |
    | x0 | First point |
    | f0 | First function value, f(x0) |
    | df0 | First derivative value, f'(x0) |
    | x1 | Second point |
    | f1 | Second function value, f(x1) |
    | df1 | Second derivative value, f'(x1) |
    | loX | Lower bound on the interval of solutions |
    | hiX | Upper bound on the interval of solutions |

Definition at line 104 of file bfgs\_linesearch.hpp.

template<typename M >

|  |  |  |  |
| --- | --- | --- | --- |
| double stan::optimization::lp\_no\_jacobian | ( | const M & | *model*, |
|  |  | std::vector< double > & | *params\_r*, |
|  |  | std::vector< int > & | *params\_i*, |
|  |  | std::ostream \* | *o* = `0` |
|  | ) |  |  |

Definition at line 288 of file bfgs.hpp.

template<typename M >

|  |  |  |  |
| --- | --- | --- | --- |
| double stan::optimization::newton\_step | ( | M & | *model*, |
|  |  | std::vector< double > & | *params\_r*, |
|  |  | std::vector< int > & | *params\_i*, |
|  |  | std::ostream \* | *output\_stream* = `0` |
|  | ) |  |  |

Definition at line 34 of file newton.hpp.

template<typename FunctorType , typename Scalar , typename XType >

|  |  |  |  |
| --- | --- | --- | --- |
| int stan::optimization::WolfeLineSearch | ( | FunctorType & | *func*, |
|  |  | Scalar & | *alpha*, |
|  |  | XType & | *x1*, |
|  |  | Scalar & | *f1*, |
|  |  | XType & | *gradx1*, |
|  |  | const XType & | *p*, |
|  |  | const XType & | *x0*, |
|  |  | const Scalar & | *f0*, |
|  |  | const XType & | *gradx0*, |
|  |  | const Scalar & | *c1*, |
|  |  | const Scalar & | *c2*, |
|  |  | const Scalar & | *minAlpha* |
|  | ) |  |  |

Perform a line search which finds an approximate solution to:

satisfying the strong Wolfe conditions: 1)  2)  where  is the gradient of f(x).

Template Parameters
:   |  |  |
    | --- | --- |
    | FunctorType | A type which supports being called as ret = func(x,f,g) where x is the input point, f and g are the function value and gradient at x and ret is non-zero if function evaluation fails. |

Parameters
:   |  |  |
    | --- | --- |
    | func | Function which is being minimized. |
    | alpha | First value of  to try. Upon return this contains the final value of the . |
    | x1 | Final point, equal to . |
    | f1 | Final point function value, equal to . |
    | gradx1 | Final point gradient, equal to . |
    | p | Search direction. It is assumed to be a descent direction such that . |
    | x0 | Value of starting point, . |
    | f0 | Value of function at starting point, . |
    | gradx0 | Value of function gradient at starting point, . |
    | c1 | Parameter of the Wolfe conditions.  Typically c1 = 1e-4. |
    | c2 | Parameter of the Wolfe conditions.  Typically c2 = 0.9. |
    | minAlpha | Smallest allowable step-size. |

Returns
:   Returns zero on success, non-zero otherwise.

Definition at line 225 of file bfgs\_linesearch.hpp.


---

[ Stan Home Page ]

*© 2011–2014,
Stan Development Team.*
